# Clinical Evaluation of Corneal Biomechanics following Laser Refractive Surgery in Myopic Eyes: A Review of the Literature

**DOI:** 10.3390/jcm12010243

**Published:** 2022-12-28

**Authors:** Zofia Pniakowska, Piotr Jurowski, Joanna Wierzbowska

**Affiliations:** 1 Optegra Eye Clinic, 90-127 Lodz, Poland; 2 Department of Ophthalmology and Vision Rehabilitation, Medical University of Lodz, 90-549 Lodz, Poland; 3 Department of Ophthalmology, Military Institute of Medicine—National Research Institute in Warsaw, 04-141 Warsaw, Poland; 4 Optegra Eye Clinic, 02-366 Warsaw, Poland

**Keywords:** laser vision correction, PRK, LASEK, LASIK, SMILE, corneal biomechanics, corneal hysteresis, corneal resistance factor, ocular response analyzer, Corvis ST

## Abstract

The role of corneal biomechanics in laser vision correction (LVC) is currently being raised in the assessment of postoperative corneal ectasia risk. The aim of the paper was to evaluate the changes in corneal biomechanics after LVC procedures based on a systematic review of current studies. The results of a search of the literature in the PubMed, Science Direct, Google Scholar, and Web of Science databases were selected for final consideration according to the PRISMA 2020 flow diagram. Included in our review were 17 prospective clinical studies, with at least 6 months of follow-up time. Corneal biomechanical properties were assessed by Ocular Response Analyzer (ORA), or Corvis ST. The results of the study revealed the highest corneal biomechanics reduction after laser in situ keratomileusis (LASIK) followed by small incision lenticule extraction (SMILE) and surface procedures, such as photorefractive keratectomy (PRK) or laser-assisted sub-epithelial keratectomy (LASEK). In SMILE procedure treatment planning, the use of thicker caps preserves the corneal biomechanics. Similarly, reduction of flap thickness in LASIK surgery maintains the corneal biomechanical strength. Future prospective clinical trials with standardization of the study groups and surgical parameters are needed to confirm the results of the current review.

## 1. Introduction

Corneal laser refractive surgery, also called laser vision correction (LVC), is a group of procedures for the correction of refractive errors, such as myopia, hyperopia, astigmatism, or presbyopia. In most cases, refractive surgery gives the possibility of complete independence from glasses or contact lenses, which significantly improves the overall patient’s quality of life. Commonly used techniques of refractive surgery are surface techniques, such as photorefractive keratectomy (PRK) or laser-assisted sub-epithelial keratectomy (LASEK) as well as stromal techniques including laser in situ keratomileusis (LASIK) and small incision lenticule extraction (SMILE). Corneal ectasia is a very rare but serious complication of refractive procedures the prevalence of which arises in 0.04–0.6% of cases [1,2]. In order to minimize the risk of its occurrence, currently the most important role is assigned to the correct qualification of the patient for laser vision correction. The preoperative analysis of the corneal structure includes pachymetry, topography, keratometry, aberrometry, and finally, corneal biomechanics, the role of which is currently being raised [3]. Understanding corneal biomechanical properties is crucial in the preoperative screening of refractive surgery candidates, to minimize the risk of postoperative corneal ectasia. Currently, there are two commercially available devices that are used in clinical practice to assess corneal biomechanical parameters; the Ocular Response Analyzer (ORA) and Corvis ST. In our study, we aimed to perform a review of the literature on changes in corneal biomechanics after laser vision correction.

## 2. Materials and Methods

The study was designed according to the PRISMA 2020 flow diagram for new systematic reviews, which includes searches of databases and registers only. 

In the systematic review, the results of a literature search on corneal biomechanics after LVC procedures were included from the following databases: PubMed, Science Direct, Google Scholar, and Web of Science. The following terms and phrases were used in database searching strategy: (corneal biomechanics OR corneal hysteresis OR corneal resistance factor OR ocular response analyzer OR ORA OR Corvis ST) AND (laser refractive surgery OR small incision lenticule extraction OR SMILE OR femtosecond lenticule extraction OR FLEX OR laser in situ keratomileusis OR LASIK OR femtosecond laser in situ keratomileusis OR femtosecond LASIK OR FemtoLASIK OR FS-LASIK OR photorefractive keratectomy OR PRK OR laser-assisted sub-epithelial keratectomy OR LASEK) NOT (review OR meta analysis). Only full-text studies published in the English language were taken into consideration. Abstracts or conference posters were excluded. The extracted publications were screened by two of the authors; Z.P. and J.W. In selecting the literature, thefocus was on the corneal hysteresis and corneal resistance factor provided by ORA as well as the dynamic corneal biomechanical response parameters measured by Corvis ST. The overview of abstracts, and subsequently the full text of articles resulted in the final selection of literature consistent with the topic. In the final selection of articles, only papers that meet the inclusion criteria, and not the exclusion criteria, were considered eligible. The following inclusion criteria were applied: prospective clinical trials, at least 6 months follow-up, refractive surgery method used (any of the following): PRK, LASEK, SMILE, LASIK, FS-LASIK, clinical assessment of corneal biomechanical properties by ORA and/or Corvis ST. The exclusion criteria were: animal study, experimental study, mathematical model, case report, letter to the author and author reply, editorial, correspondence, conference abstract, review, meta-analysis, systematic review, not English. Questionable cases were discussed in detail by the authors with their final consensus.

**Figure 1:**
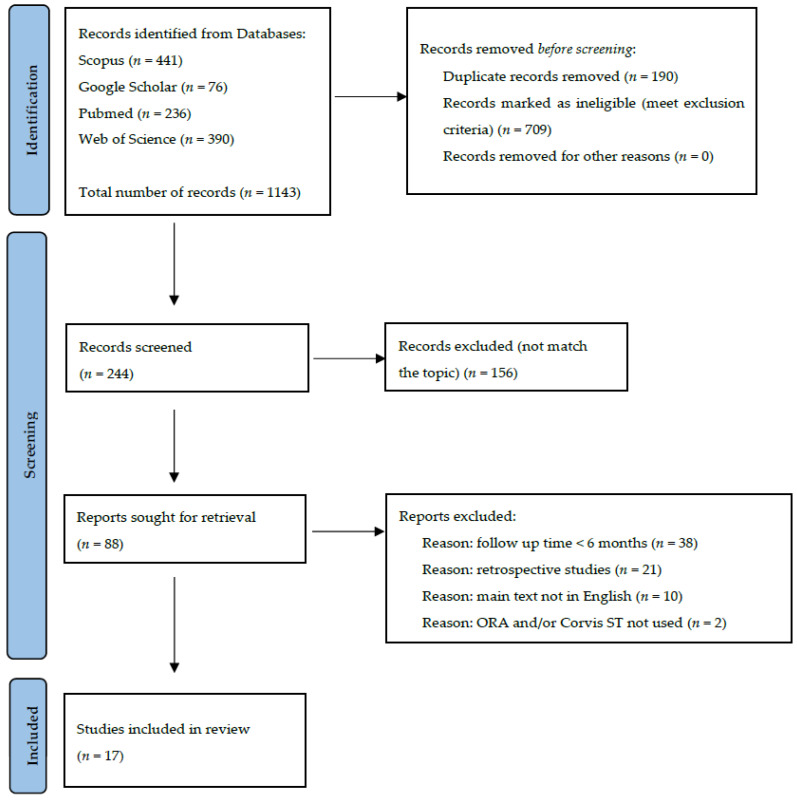
Prisma 2020 flow diagram for Identification of studies via databases.

The literature selection process for the systematic review is presented in the PRISMA 2020 flow diagram (Figure 1). The preliminary number of all database search results was 1143 papers. Among the records removed before screening, there were 709 records marked as ineligible and 190 duplicated articles; 244 results were screened. In the screening process, 150 articles were excluded, being outside the topic of our study, 16 papers were not available as full-text articles, 28 articles did not meet follow-up time criteria, 21 were retrospective studies, 10 were not in English, and 2 studies did not include ORA and/or Corvis measurements. Finally, we included 17 studies that were eligible for our systematic review.

The most frequently used technologies in the clinical evaluation of corneal biomechanics are the corneal response technology used in the Ocular Response Analyzer (ORA; Reichert Ophthalmic Instruments, NJ, USA) and the Scheimpflug camera technology of Corvis ST—the Dynamic Scheimpflug Tonometer (DST; CST; Oculus Optic-gerate, Inc. Wetzlar, Germany). Both devices provide in vivo measurements of corneal biomechanical parameters and the corneal-corrected intraocular pressure. 

Corneal hysteresis (CH) is defined as the difference between two applanation pressures P1 and P2 obtained during corneal deflection by the air-impulse generated by an ORA tonometer. Clinically, CH is a measure of the viscoelasticity of the cornea, meaning the time-dependent response of the corneal tissue to the applied force of an air puff. Corneal resistance factor (CRF) is described mathematically as P1 − K × P2, where K = 0.7, being an indicator of a static, time-independent corneal response to the applied force. In practice, CRF is a measure of the elasticity of the cornea taking into account its central thickness, which reflects the total resistance (stiffness, strength) of the cornea. In addition, ORA provides 2 values of intraocular pressure (IOP): Goldmann-correlated IOP (IOPg) and corneal-compensated IOP (IOPcc) [4,5,6].

Dynamic Scheimpflug Analyzer Corvis ST is a non-contact tonometer which analyses the corneal biomechanics based on the corneal deformation by an air pulse. The device takes 140 horizontal 8 mm frames in 33 ms, allowing capture of the corneal deflection in applanation points [7]. The mathematical analysis of corneal behavior during the measurement provides the specific parameters of overall corneal biomechanics. The deformation amplitude (DA) refers to the inward dislocation of the corneal apex measured at the highest concavity (HC) point [7]. The DA ratio of central and peripheral deflection is measured at a distance of 1 mm and 2 mm resulting in DAR-1 and DAR-2 parameters and a stiffness parameter at the 1st applanation (SPA1) [8]. Applanation lengths (AL) and corneal velocities (CVel) are measured during the inward and outward corneal deformation. The other parameters assessed by Corvis are the curvature radius at the highest concavity (curvature radius HC), the integrated inverse radius (IntInverseR), and maximum inverse radius (InverseR). The higher the value of IR, the lower the corneal resistance to deformation which indicates lower corneal stiffness [8]. The Ambrosio Rational Thickness horizontal (ARTh) and Pachyslope, together with Corneal pachymetry (Pachy), are significant parameters in the screening for keratoconus [8]. To define the difference between healthy corneas and those with subclinical ectasia or keratoconus, the Dynamic Corneal Response (DCR) was combined with the Corvis biomechanical index (CBI) and tomographic and biomechanical index (TBI) in the Corvis software [8]. The biomechanical corrected intraocular pressure (bIOP) is the value of IOP minimally affected by corneal biomechanics [9].

## 3. Results

### 3.1. Surface Ablation

PRK is a surface LVC procedure in which the corneal epithelium is carefully removed. Subsequently, the anterior stroma is ablated by the excimer laser. The epithelial abrasion is related to postoperative pain in the vast majority of patients [3]. LASEK is another surface procedure in which the corneal epithelium is not totally removed, but detached after soaking with 20% ethanol, then pushed aside just before anterior stroma ablation. The epithelium is then slid over the cornea again, which reduces postoperative pain [3].

Six studies that reported an influence of surface procedures on corneal biomechanics were included in our review. In four papers, ORA was used to measure CH and CRF while in two studies, the authors used Corvis ST (Table 1).

The prospective, single-center study by Ryan et al., included 51 eyes of 51 myopic and myopic astigmatism patients, who were treated with bilateral epi-LASIK [10]. The mean preoperative spherical equivalent refraction (MRSE) was 2.68 ± 1.08 Diopters (D). Epikeratome was used for the corneal de-epithelialisation, with subsequent ablation of the corneal stroma by excimer laser. CH and CRF were measured by ORA and central corneal thickness was measured using an ultrasound pachymeter preoperatively and at 1-, 3-, 6-, and 12-month follow-ups. Mean CH and CRF values were significantly reduced over time at each follow-up point (Table 2). One-way repeated measures ANOVA demonstrated that CH and CRF changed over time (F_3.0, 118.1_ = 96.1, *p ≤* 0.0005; F_3.2, 128.4_ = 91.3, *p* ≤ 0.0005 respectively) as a result of epi-LASIK, while the significant decrease in CH and CRF values were observed only in the first postoperative month [10].

In the study by Xin et al. [11], the impact of t-PRK, FS-LASIK, and SMILE on corneal biomechanical changes was assessed over a 6-month follow-up period. A total of 227 myopic patients with a mean of −4.82 ± 1.57 D and astigmatism mean of −0.76 ± 0.59 D were included in the study. 74 patients underwent t-PRK, 81 FS-LASIK, and 72 underwent SMILE. In the FS-LASIK group, a 95 to 110 µm-thick flap with a diameter from 8.5 to 9.0 mm and a superior hinge was created. The cap thickness in the SMILE procedure ranged from 115 to 140 µm. Corvis ST was used to measure the specific corneal biomechanical parameters: SP-A1, IIR, DA, and DAR-2 (Table 3). The cornea’s overall stiffness was significantly reduced in all study groups. The t-PRK was associated with the least stiffness reduction, followed by SMILE, and then FS-LASIK which compromised corneal biomechanics the most [11].

Hwang et al. [12] assessed the influence of PRK without mitomycin C (MMC), PRK with MMC, and LASIK on CH and CRF. A total of 194 eyes completed a 12-month follow-up. In eyes with myopia above −6.0 D and cylinder above 3.0 D, 0.02% MMC was applied for 15 s. LASIK flaps were created with an intended thickness of 110 µm and 8.8 mm diameter. The assessment of corneal biomechanical parameters was performed at baseline and at 3 months, 6 months, and 12 months postoperatively. Three months after surgery, CRF was significantly reduced in eyes that underwent LASIK, PRK without MMC, and PRK with MMC. The study revealed that eyes that had PRK with MMC showed the greatest decrease in CRF and CH in the first 3 months of follow-up while from the 3rd to the 12th postoperative month, the corneal biomechanical strength increased significantly. In all studied LVC procedures (LASIK, PRK without MMC, and PRK with MMC), a significant decrease in corneal biomechanics was reported. There were, however, no differences between groups in terms of postoperative corneal biomechanics weakening [12].

In the study by Qazi et al. [13], 30 eyes were selected for surface ablation - myopic LASEK (mLASEK) group, and 28 eyes were included in the myopic LASIK (mLASIK) group. Primarily, eyes with thinner preoperative CCT were recruited for the mLASEK group, and a 9.0 mm diameter ring was applied on the corneal surface and filled with ethanol 20% for 20 s. Then, the epithelial flap (50 μm deep) was created to expose the corneal stroma. After the stromal ablation, a bandage contact lens was applied in low myopic patients. In patients with myopia above −6.0 D or stromal ablation above 100 μm, a circular sponge soaked with mitomycin-C 0.01% was applied on the ablation zone for 20 s, followed by thorough irrigation with balanced salt solution (BSS). In the mLASIK group, a superiorly hinged and 160–180 μm thick lamellar flap was created with microkeratome. In all participants, corneal biomechanical properties, such as CH and CRF were measured by ORA. The mean postoperative CH was significantly lower in the mLASEK group than in the mLASIK group. The CRF was postoperatively lowered in the mLASEK and mLASIK groups, while there was no difference in this parameter between those groups (Table 2).

In the prospective study by Yu et al., 64 patients were enrolled who had undergone LASEK (32 eyes) or SMILE (32 eyes) [14]. In the LASEK group, an epithelial flap was created with 20% ethanol applied for 14 s then peeled back with a crescent blade. The 4–6-mm wide hinge was located superiorly. Corneal stromal tissue ablation was performed using a Mel 80 excimer laser without post-ablation MMC. In the SMILE group, the cap parameters were as follows: 120 μm thickness, 7.5 mm diameter, and 2.1 mm side cut length at the superior position. The corneal biomechanics were assessed with ORA at 3-months and 3-year post-operation control points. The post-operative CH and CRF were significantly lower than the preoperative values in both groups (*p* < 0.01). In the SMILE group, CH and CRF were stable in observation time between 3 months and 3 years post-operation. CH was higher in SMILE than in the LASEK group 3 months postoperatively (CH: *p* = 0.03). In turn, the CH parameter increased in the LASEK group (CH: *p* = 0.001) 3 years after surgery. The decrease of CH and CRF per unit of corneal tissue removed was less in SMILE than in LASEK at 3 months after surgery. However, three years after surgery, the impact of SMILE and LASEK on the corneal biomechanical strength was similar. The authors concluded that the decrease in CRF and CH per unit of tissue removal was less after SMILE than LASEK when assessed in the early postoperative period as well as during long-term observation (Table 2). 

Hashemi et al., presented the biomechanical strength measured by Corvis ST in patients who had undergone PRK-MMC or FS-LASIK [15]. Thirty myopic patients in each group with MRSE greater than −7.0 D were included in this nonrandomized comparative study. In the PRK group, after mechanical epithelium removal and surface ablation, MMC 0.02% (0.2 mg/mL) was applied on the corneal stroma for 10 s per diopter of ablation. In the FS-LASIK group, a 9.5 mm diameter and 110 mm deep flap was created with the superior hinge location. Corvis ST corneal biomechanical parameters were measured at 3 and 6 postoperative months. In the PRK-MMC group, the authors observed a significant rise in A2-time, A2-velocity, and PD (Table 3). Simultaneously, a significant decrease was reported in A1-time, A1-velocity, and radius (*p* ≤ 0.05 for all parameters). In turn, there was no significant postoperative change in A1-length, A2-length, highest concavity time, and DA when measured 6 months postoperatively. In the FS-LASIK group, an increase was reported in A2-time, A2-velocity, and DA, and a decrease in IOP, corrected IOP, A2-length, A1-velocity, and radius (*p* ≤ 0.05 for all parameters). No significant difference was observed in A1 length, highest concavity time, and PD parameters. The authors concluded that there was a significant change in corneal biomechanics measured by Corvis ST after both FS-LASIK and PRK-MMC refractive surgeries in myopia correction. The biomechanical changes were more prominent in the FS-LASIK group for a 6-month follow-up period [15]. The general means of corneal biomechanical parameters, in the surface ablation studies, were as follows: CH 8.68 ± 0.94, CRF 8.39 ± 1.08, A1T 6.55 ± 0.21, SP A1 71.25 ± 4.60, DA 1.13 ± 0.05.

### 3.2. LASIK and FS-LASIK 

In a LASIK procedure, the corneal ablation is performed in deeper stroma, which must be preceded by the corneal flap formation. The flap formation technique was previously based on the use of microkeratomes, but currently, corneal flaps in LASIK are mostly formed by femtosecond laser. The LASIK procedure is, therefore, called femtosecond LASIK (FS- LASIK) [3].

In this review, 11 articles addressed changes in corneal biomechanics after LASIK/FS-LASIK surgery. In 7 of these studies, biomechanical properties of the cornea were assessed by ORA, and in the other 4 studies, Corvis ST was used (Table 1).

Yang et al., compared the corneal biomechanics measured by Corvis ST in 23 normal eyes, 23 LASIK eyes, 23 KE eyes and 23 KC eyes [16]. The inclusion criteria for the LASIK group were state at least 1 year post LASIK surgery, (BCVA) LogMAR ≤ 0.1, and no presence of corneal ectasia in the topographic maps. The authors reported that Max Inverse Radius, DAR-2, Pachy Slope, Dar-1, IR, and CBI in LASIK eyes, KE eyes, and KC eyes were higher than in normal eyes. In turn, the ARTh and SP-A1 parameters were lower in the LASIK, KE, and KC groups than in normal eyes. In KE eyes, the mean values of Max IR, DAR-2 mm, Pachy Slope, DAR-1, and IR were significantly higher than in LASIK eyes. In contrast, the SP-A1 value was lower in the KE group than in the LASIK group. The area under the ROC curve of IR, Max IR, DAR-2, and SP-A1 was above 0.800, allowing KE to be distinguished from post-LASIK eyes. The authors concluded that the corneal biomechanical parameters measured by Corvis ST might be helpful in distinguishing KE eyes from LASIK eyes. However, there is a necessity for further studies to confirm the author’s findings [16].

The prospective study of Wu Di et al., concerned the changes in CH, CRF, and 37 other biomechanical waveform parameters after FS-LASIK and SMILE procedures [17]. Corneal biomechanics were assessed by ORA preoperatively as well as 1 week and 1, 3, and 6 months postoperatively. The flap parameters in the FS-LASIK group were 7.9–8.0 mm in diameter and 100–110 μm thickness. Each study group comprised 40 eyes. At most postoperative follow-up points, the values of CH and CRF were significantly decreased in the FS-LASIK and SMILE groups (*p* < 0001) (Table 2). In the SMILE group, the CH and CRF values were significantly higher than in the FS-LASIK group when measured at each time point of follow-up. The authors concluded that, although both LVC procedures reduce the corneal biomechanics, the change in the CH and CRF was smaller and showed better predictability after SMILE than after FS-LASIK [17].

In the report by Elmohamady et al., 103 eyes with myopia from −3.00 to −10.00 and astigmatism less than 4 D were recruited for LVC [18]. A LASIK procedure was performed on 30 eyes, FS-LASIK on 38 eyes, and SMILE surgery on 35 eyes. A 100 µm corneal flap was created using Moria microkeratome in the LASIK group, while in the FS-LASIK group, a femtosecond VisuMax laser was used for 100 µm flap creation. The stromal ablation was done with a Meditec MEL90 excimer laser. In the SMILE group, the microlenticule was created by the VisuMax femtosecond laser. Postoperative evaluation of corneal biomechanical parameters was performed with the use of an ocular response analyzer. Corneal hysteresis (CH) and corneal resistance factor (CRF) were assessed at 1, 3, 6, 12, 24, and 36 months after surgery. The authors reported that both CH and CRF parameters were significantly reduced postoperatively as compared to preoperative values in all study groups. In the SMILE group, CH and CRF were significantly higher than in the LASIK and FS-LASIK groups at any follow-up point (*p* ≤ 0.05) (Table 2). 

In their prospective observational study, Vanathi et al., evaluated the influence of myopic FS-LASIK on corneal biomechanical changes and their correlation with the percentage of tissue altered (PTA) [19]. The authors included 80 myopic eyes with mean MRSE −3.5 ± 1.6 D, treated with 120 μm-flap FS LASIK. The corneal biomechanical parameters, such as CH and CRF were assessed by ORA preoperatively and at specific postoperative points (day 1 and month 1, 3, and 6). The study group was divided into three subgroups [subgroup 1: PTA 23 to <27%, subgroup 2: 27 to <33%, subgroup 3: 33 to 40%]. In all study groups, there was a decrease in CH and CRF after surgery when measured at each follow-up point. CH and CRF correlated negatively with PTA (CH: r = −0.33 [*p* ≤ 0.0001], CRF: r = −0.34 [*p* ≤ 0.001] (Table 2). The authors concluded that myopic FS-LASIK causes a significant decrease in corneal biomechanical parameters. A greater decrease in CH and CRF was reported in eyes with higher PTA (subgroup 3) [19]. 

Agca et al., assessed sixty eyes of 30 patients with bilateral myopia (MRSE) <10 dioptres (D) and a difference in MRSE ≤ 0.50 D in both studied eyes [20]. In one eye the SMILE procedure was performed, while the fellow eye was treated with FS-LASIK. The CH and CRF were measured preoperatively and at 1 month and 6 months postoperatively. The authors reported that CH and CRF values were significantly reduced postoperatively in both groups in comparison with the preoperative values (*p* < 0.001) (Table 2). There were, however, no differences in postoperative CH or CRF values when measured in both the SMILE and FS-LASIK groups [20]. 

Thirty-six myopic eyes with MRSE –4.39 ± 1.43 D, treated with LASIK surgery, were included in a study by Kamiya [21]. The LSK-1 microkeratome was used to create a 130 μm- thick corneal flap. The biomechanical parameters of the cornea, such as CH and CRF were assessed by ORA preoperatively and at 1 week and 1, 3, and 6 months after the LASIK procedure. The mean preoperative value of CH was 10.6 ± 1.7 mm Hg, and CRF was 10.0 ± 1.7 mm Hg. Statistical analysis revealed a significant decrease in CH and CRF values when measured at 1 week and 1, 3, and 6 months postoperatively (Table 2). The authors concluded that the LASIK procedure weakened the biomechanical properties of the cornea irreversibly in the early postoperative period. The biomechanical parameters, however, had stabilized over the postoperative 6 months with no further decrease [21]. 

In the prospective comparative contralateral eye study by He et al., 50 patients with high myopia (MRSE greater than −6.0 D) were treated with SMILE in one eye and FS-LASIK in the fellow eye [22]. The authors aimed to compare the corneal biomechanical properties of the treated corneas measured by Corvis ST before and at 1, 3, 6 months and more than 1 year after surgery. The differences in SE and cylinder between the paired eyes ≤1 D, and postoperative RST was greater than 280 μm for SMILE and 300 μm for FS-LASIK. The following cap parameters were used in the SMILE procedure: thickness 110 µm and diameter from 7 to 7.7 mm. In the FS-LASIK, the VisuMax femtosecond laser was used to create a 95 µm-thick flap of a diameter from 8.1 mm to 8.5 mm. ARTh and SP-A1 parameters decreased significantly after SMILE and FS-LASIK, while the postoperative DAR-2 and IR increased (Table 3). The ARTh and SP-A1 parameters were significantly greater following SMILE than FS-LASIK at all the follow-up points (*p* < 0.05). Other biomechanical parameters did not differ significantly between the study groups. Moreover, the biomechanical parameters of treated corneas in both study groups were stable from 1 to 15 postoperative months. The authors concluded that the SMILE procedure in high myopia cases may compromise the corneal biomechanics less than FS-LASIK with similar MRSE correction [22]. The general means of corneal biomechanical parameters, in the LASIK and FS-LASIK studies, were as follows: CH 8.46 ± 0.85, CRF 7.77 ± 0.96, A1T 6.99 ± 0.18, SP A1 69.91 ± 6.08, DA 1.10 ± 0.13.

### 3.3. SMILE

The invention of the femtosecond laser led to the development of new techniques for laser vision correction, such as SMILE. In the SMILE procedure, the femtosecond laser creates a lenticule in the corneal stroma and then side cuts in the peripheral cornea to allow mechanical removal of the lenticule through a small side cut [3]. The LASIK, FS- LASIK, and SMILE procedures provide a painless healing process and relatively quick visual recovery.

A total of 10 studies included in our review presented the corneal biomechanical outcomes after SMILE surgery. The biomechanical parameters were assessed by ORA in 6 studies, while Corvis ST was used in 4 reports (Table 1). 

In the first comparative, contralateral eye study, Wu De et al., assessed the effect of cap thickness applied on corneal biomechanical parameters measured with Corvis ST following the SMILE procedure. [23]. The study groups were comprised of 100 eyes (50 eyes in each group) treated for myopia with a 110 μm cap or 140 μm cap thickness SMILE procedure. The difference between eyes in MRSE was <0.50 D. The postoperative assessment of corneal biomechanical parameters was performed on the 1st day and in the 1st month, 3rd month and 6th month after the surgery. Six months after the surgery, there were significant changes in all corneal biomechanical parameters after SMILE except for the time until the highest concavity. Significant differences were found between study groups in AT2, integrated radius, and DA when measured 6 months postoperatively (Table 3). Moreover, the changes in AT2, DA, and integrated radius were smaller in the 110-µm cap group than in the 140-µm cap group. At each follow-up point, the change in curvature of the anterior surface in the 4-mm zone was greater in the 110-µm cap thickness group than in the 140 μm cap group, which suggests that cap thickness affects the change in curvature of anterior corneal surface. The authors concluded that the use of a thicker corneal cap leads to less change in anterior corneal curvature, which is not related to either visual acuity or refractive outcomes [23]. 

In a study by Wu Z et al., the influence of microincision lenticule extraction (MILE) and SMILE procedures on the change in corneal biomechanics was assessed [24]. Sixty myopic eyes were treated using the MILE method with a 2 mm side cut, while 64 eyes received SMILE treatment with a 5 mm side cut. In both study groups, cap parameters were a thickness of 110 μm and a diameter of 6.2 mm. CH, CRF, and 37 biomechanical waveform parameters were assessed with the use of ORA preoperatively and 1 week, 1 month, and 6 months postoperatively. Both CH and CRF values decreased significantly after MILE and SMILE (*p* < 0.05). Although CH decreased significantly 1 week after surgery, a gradual increase was observed in this parameter from 1 to 6 months postoperatively in both study groups. CRF measured at the 1st postoperative week was statistically lower than at 1 month (*p* = 0.001) (Table 2). Finally, there were no significant differences between the MILE and SMILE study groups in terms of the postoperative values of CH and CRF [24].

Jun et al., compared the corneal biomechanical changes after SMILE with 120-μm and 140-μm cap thickness [25]. A total of 150 eyes (91 eyes in the 120-μm group and 59 eyes in the 140-μm group) with myopia <8.00 D were included in the study. SMILE was performed with the use of a VisuMax femtosecond laser. Corneal biomechanical properties were measured with use of Corvis ST. The postoperative biomechanical results revealed that the DA ratio and IIR increased significantly, whereas there was a significant reduction of SP-A1, ARTh, and SSI parameters. The above findings indicate a significant decrease in corneal stiffness and resistance to deformation. In addition, a significant difference was observed in the pre- and postoperative values of the DA ratio and IIR between the two study groups, which suggests that the corneal biomechanical strength was greater in the 120-μm cap group than in the 140-μm cap group (*p* = 0.022 for DA ratio, and *p* = 0.011 for the IIR) (Table 3). In summary, the authors stated that the thicker lenticule required for a thick cap may contribute to a greater weakening of corneal biomechanics, which is associated with a thinner RSB. Further studies are required to better understand the effects of cap thickness on corneal biomechanics [25].

A study by Vestergaard et al., compared corneal biomechanical properties in patients with myopia (SE −6.00 to −10.00 D) who had undergone FLEX or SMILE surgery [26]. The flap/cap diameter was 7.9 to 8.0 mm in FLEX and 7.3 mm in SMILE. The intended flap/cap thickness was 100 to 120 µm. Corneal biomechanics were assessed by ORA. CH and CRF values were reduced by 2.7 ± 1.3 mm Hg (*p* < 0.01) and 4.5 ± 1.2 mm Hg (*p* < 0.01), respectively when measured at 6 months after FLEX. Similarly, following SMILE, CH and CRF were significantly reduced (3.3 ± 1.2 mm Hg, *p* < 0.01; 4.6 ± 1.2 mm Hg, *p* < 0.01, respectively). Additionally, there was no statistically significant difference between both study groups in terms of postoperative change in CH (*p* = 0.08) and CRF (*p* = 0.71) (Table 3). The results of the study suggest that the surgically induced changes in corneal biomechanical parameters were independent of the treatment method (FLEX or SMILE) up to 6 months of follow-up after the surgery [26]. The general means of corneal biomechanical parameters, in the SMILE studies, were as follows: CH 8.41 ± 0.38, CRF 7.36 ± 0.65, A1T 7.09 ± 0.19, SP A1 78.57 ± 11.00, DA 1.19 ± 0.01.

## 4. Discussion

Currently, it is well known that corneal refractive surgery compromises corneal biomechanics [3,4,9,10,11,12,13,14,15,16,17,18,19,20,21,22,23,24,25,26,27,28,29,30,31,32]. Nevertheless, the impact of specific LVC techniques on corneal biomechanical properties is currently under research. Our systematic review of the literature comprises 17 prospective studies on changes in corneal biomechanical properties following refractive surgery procedures (trans-PRK, PRK with MMC, LASEK, epi-LASIK, LASIK, FS-LASIK, FLEX, SMILE, and MILE).

### 4.1. Corneal Biomechanics after PRK/LASEK versus FS- LASIK or SMILE

The results of recent studies comparing corneal biomechanics after surface procedures (PRK with or without MMC, trans-PRK, LASEK) versus FS- LASIK are mostly consistent. The authors conclude that surface procedures weaken the biomechanics of the cornea less than or at least equal to LASIK or FS-LASIK [11,12,13,15,33,34,35]. There are, however, controversies over corneal biomechanics after surface procedures in comparison with SMILE [34]. The meta-analysis by Guo revealed that ORA parameters CH and CRF were insignificantly higher after PRK or LASEK than after SMILE [3]. However, in most studies that were taken into consideration, the amount of tissue removed was greater in the SMILE procedure than in PRK/LASEK, as the SMILE was performed to treat higher myopia than surface procedures [3]. As the authors concluded, to obtain more reliable results in the comparison of corneal biomechanics after surface procedures and SMILE, future studies should take into account the degree of myopia as an important inclusion criterion in research groups [3]. An attempt at the unification of study groups in terms of the degree of refractive error was made by Yu et al., who compared the CH and CRF per unit of corneal tissue removal after SMILE and LASEK, concluding that corneal biomechanics were stronger after SMILE than after LASEK in the early postoperative period [14]. Nevertheless, during long-term observation, the difference became insignificant [14]. The comparison of corneal biomechanics between SMILE and LASEK eyes with Corvis ST was performed by Shen et al., who found no differences between study groups [36].

### 4.2. Corneal Biomechanics after SMILE versus FS-LASIK or FLEX

In the vast majority of studies included in this review, the postoperative biomechanical outcomes were better following SMILE than after LASIK or FS-LASIK [11,17,18,22]. Similarly, in the meta-analysis by Guo et al., CH and CRF provided by ORA were significantly higher after SMILE than after FS-LASIK or microkeratome-LASIK. Moreover, the difference increased when measured at 12 months postoperatively in favor of SMILE [3]. This is also consistent with the outcomes of a meta-analysis by Yan et al., of 5 studies in which the CH and CRF values were higher after SMILE than FS-LASIK [37]. Wang et al., assessed changes in posterior corneal elevation and corneal biomechanical parameters after SMILE and FS-LASIK for high myopia correction [38]. Their study revealed that SMILE maintained posterior corneal surface stability better than FS-LASIK at 12 months after surgery [38]. Corneal biomechanical parameters were similar after the two procedures, although FS-LASIK led to a greater reduction of postoperative CRF. The authors concluded that SMILE could be more advantageous biomechanically, particularly in high myopia correction [38]. In the review study by Raevdal et al., including six non-randomized and three randomized control trials, the authors found a significant reduction of corneal viscoelastic properties measured by ORA following all types of refractive procedures (SMILE, FLEX, FS-LASIK) [39]. The authors of the six non-randomized studies reported greater postoperative reduction of corneal biomechanics in the FS-LASIK group than in the SMILE group when measured by ORA [39]. In contrast, in the three randomized control trials included in their meta-analysis [39], no significant difference was found in terms of CH or CRF lowering between SMILE and flap- related procedures (FLEX or FS-LASIK) [20,26,40,41]. This was also consistent with the studies by Vestargaard et al. [20] and Agca et al. [26], which revealed no biomechanical differences between SMILE and FS-LASIK or FLEX procedures. [20,26]. Similar data were provided in the meta-analysis by Guo et al., in which corneal biomechanics did not differ after SMILE or FLEX procedures when measured either by ORA or Corvis ST [3]. In several studies included in this review and also mentioned by Raevdal et al. [39], the corneal parameters were assessed by Corvis ST and revealed a significant weakening of corneal biomechanics after refractive procedures [11,15,16,22,23,25,42,43]. There, are, however controversies in terms of Corvis ST biomechanics post SMILE and FS-LASIK. Studies by Xin et al. [11] and He et al. [22] demonstrated significantly better corneal biomechanics preservation following SMILE than after LASIK or FS-LASIK, when assessed by Corvis ST [11,22]. On the other hand, in the majority of studies included in the meta-analysis by Guo et al. [3], the postoperative biomechanical parameters measured with Corvis ST did not differ between SMILE and FS-LASIK eyes [3]. Only in the study by Osman were there significant differences in the biomechanical parameters provided by Corvis ST (A1time, A2 time, A2 length, HC time, HC radius, and HC peak distance) between the SMILE group and microkeratome-LASIK group [44]. The authors concluded that the reason for this dissimilarity of results could be the use of microkeratome for flap creation [44]. Damgaard et al. [4] observed that the corneal biomechanical parameters provided by Corvis ST and ORA were stronger, or at least equal, after SMILE rather than LASIK. However, the authors stated that although the precise values of corneal biomechanical properties are provided by ORA and Corvis ST, the interpretation of biomechanical results is mostly biased by IOP, CCT, the magnitude of corrected refractive error, and age [4]. Kanellopoulos et al., demonstrated that the SMILE procedure performed in low myopic eyes resulted in greater tensile strength reduction than with the LASIK procedure, while in higher myopia, the reduction of corneal strength was similar in both SMILE and LASIK eyes [29]. Wei et al., assessed the change in biomechanical properties per unit of reduction in corneal volume, concluding that the change was lower following SMILE than FS-LASIK [45]. Therefore, future paired-eye studies and the unification of study groups are needed to confirm the biomechanical findings after SMILE versus FS-LASIK [4]. 

### 4.3. Cap or Flap Thickness and Corneal Biomechanics 

The influence of cap thickness on corneal biomechanics remains unclear. Many surgeons believe that increasing the thickness of the cap maintains stronger biomechanics of the cornea [36]. Recently, Wu et al., conducted a prospective contralateral eye study comparing the corneal biomechanics and curvature after SMILE in eyes with thinner (110 μm) and thicker (140 μm) caps [23]. The study was included in this review. In eyes with a 110 μm cap, the second applanation time (SP-A2), deformation amplitude (DA), and integrated radius were significantly lower than in eyes with a 140 μm cap [23]. Similarly, El-Massary et al., reported less corneal biomechanics weakening with a 160 μm cap than with a 100 μm cap based on the results of CH and CRF measured with ORA [46]. The above findings are consistent with the concept described by Reinstein et al. [47] and Randleman et al. [48], that corneal biomechanics and tensile strength are higher in anterior than posterior stroma, which suggests greater corneal biomechanics in thicker caps [23,25,46,47,48,49]. Although the corneal biomechanics are better preserved in thicker caps, in high myopic patients, the increase in cap thickness results in deeper lenticule creation and a thinner posterior residual stromal bed (RSB), which can finally weaken the corneal biomechanics [25]. In research by Jun et al., the corneal biomechanics were weaker in the 140 µm cap group compared to the 120 µm cap group, which was confirmed by the greater differences in the corneal shape deformation, deformation amplitude ratio, and integrated inverse radius in the 140 µm cap group [25]. However, the authors noted that the lenticule thickness was significantly greater and the RSB was significantly thinner in the 140 µm cap group in comparison to the 120 μm cap group, which might have strongly influenced the biomechanical outcomes [25]. The authors of the study postulated that the role of cap thickness in preserving corneal biomechanics should be verified in further studies. In addition, other parameters, such as the thickness of the lenticule, percentage of tissue removed, anterior and posterior residual stromal bed, the arc length of the posterior cap, and size of the side incision should be taken into account in this consideration [25].

According to Reinstein et al., for maximum protection of the corneal biomechanics, it is recommended to prepare thin corneal flaps using the LASIK or FemtoLASIK methods and a thicker cap using SMILE [50]. The significant reduction of corneal biomechanical parameters, such as CH and/or CRF was also reported by other authors who compared patients after 110 μm flap LASIK versus epi-LASIK, 130 μm flap LASIK versus PRK and finally 160 μm/180 μm flap LASIK versus LASEK [13,51,52]. In all the above-mentioned studies, the CH and/or CFR were higher after surface procedures rather than after LASIK. Medeiros et al., compared the corneal biomechanical parameters reduction after thick-flaps LASIK and thin-flaps LASIK, concluding that thick LASIK flaps compromised the corneal biomechanics much more than thinner flaps [53]. The study by Goussous et al., compared the preoperative and 3-months postoperative values of CH and CRF in patients who underwent either epi-LASIK with MMC, thin-flap (90 µm) LASIK, or thick-flap (130 µm) LASIK [54]. The greater reduction of CH and CRF was observed after both 90 μm and 130 μm flap LASIK than after epi-LASIK. CH was significantly lower in thick-flap LASIK than in epi-LASIK eyes, while the CH difference between thin-flap LASIK and epi-LASIK eyes was not statistically significant [54]. The CRF reduction in both LASIK groups compared to the epi-LASIK group was not statistically significant but the decrease in CRF value was greater in the thick-flap LASIK group than in the thin-flap LASIK group [54]. There were, however, some limitations of the study, including greater ablation depth in the 90 μm flap LASIK patients, greater preoperative CCT and CH in the 130 μm flap LASIK patients, and use of M-2 microkeratome which overcut the flaps on average 10–15 μm (no flap thickness control was performed postoperatively) [54]. To summarize, most recent clinical studies support the statement that the biomechanical strength of the cornea decreases with LASIK-flap thickness increase [13,51,52,54,55]. However, future studies are needed to obtain more reliable outcomes [54].

## 5. Conclusions

In conclusion, LASIK is associated with the largest corneal biomechanics reduction, followed by SMILE and then surface procedures, such as LASEK, PRK, or epi-LASIK. The increase of cap thickness in SMILE and decrease of flap thickness in LASIK produces less reduction in corneal biomechanics in myopic eyes following laser correction. Further long-term studies are needed to comprehensively understand the effects of both corneal and surgical LVC parameters on corneal biomechanics and to discuss the relations between the ORA and Corvis ST parameters in eyes following corneal laser refractive surgery. 

## Figures and Tables

**Table 1:** Characteristics of prospective studies included in the systematic review.

First Author, Year of Publication	Sample Size (Eyes)	Follow-Up Time (Months)	Mean Spherical Equivalent (SE)	Type of Refractive Surgery Procedure	Corneal Biomechanics Assessment Device	Corneal Biomechanics or IOP Parameters Assessed
Ryan et al., 2011 [10]	51	12	−2.68 ± 1.08 D	epi-LASIK	ORA	CH and CRF
Xin et al., 2022 [11]	74 t-PRK 81 FS-LASIK 72 SMILE	6	t-PRK −4.69 ± 1.57 D FS-LASIK −5.04 ± 1.58 D SMILE −4.72 ± 1.57 D	t-PRK, FS-LASIK, SMILE	Corvis ST	SP-A1, AdjAP1, bIOP, A1DeflAmp, IIR, DA, DAR-2
Hwang et al., 2017 [12]	194	12	−3.5 ± 2.1 LASIK −3.1 ± 1.4 PRK −5.9 ± 2.3 PRK-MMC	PRK, PRK-MMC, LASIK	ORA	CH, CRF
Qazi et al., 2009 [13]	14 LASIK 15 LASEK 29 total	6	−5.32 ± 2.70 LASIK −4.55 ± 3.03 LASEK	LASIK, LASEK	ORA	CH, CRF, IOPg, IOPcc
Yu et al., 2019 [14]	32	3 Y	−4.1 ± 0.8 SMILE −3.7 ± 1.0 LASEK	SMILE LASEK	ORA	CH, CRF, IOPg, IOPcc
Hashemi et al., 2017 [15]	60	6	−8.65 ± 1.51 D FS-LASIK −8.04 ±1.70 D PRK-MMC	FS-LASIK, PRK + MMC	Corvis ST	IOP, bIOP, A1T, A2T, A1L, A2L, A1V, A2V, HCT, DA, HC PD, HCR
Yang et al., 2020 [16]	23	12	N/S	LASIK	CORVIS- ST	IR, DAR-2, Pachyslope, DAR-1, ARTh, IIR, SP-A1, CBI
Wu Di et al., 2014 [17]	40	6	−5.71 ± 1.19 SMILE −5.80 ± 1.14 FS-LASIK	SMILE, FS-LASIK	ORA	CH, CRF, 37 biomechanical waveform parameters
Elmohamady et al., 2018 [18]	103	3 Y	−7.49 ± 2.05 LASIK −7.14 ± 1.97 FS-LASIK −8.05 ± 2.06 SMILE	LASIK, FS-LASIK, SMILE	ORA	CH, CRF
Vanathi et al., 2020 [19]	80 group 1: PTA 23 to <27%; group 2: 27 to <33%; group 3: 33 to <40%	6	−1.4 ± 0.4 group 1 −3.1 ± 1.0 group 2 −4.9 ± 1.1 group 3 −3.5 ± 1.6 total	FS-LASIK	ORA	CH, CRF, IOPg, IOPcc
Agca et al., 2014 [20]	60	6	−3.62 ± 1.79 SMILE −3.71 ± 1.83 FS-LASIK	SMILE, FS-LASIK	ORA	CH, CRF
Kamiya et al., 2009 [21]	36	6	–4.39 ± 1.43 D	LASIK	ORA	CH, CRF
He et al., 2022 [22]	50	6	−8.46 ± 1.04 SMILE −8.52 ± 1.12 FS-LASIK	SMILE, FS-LASIK	Corvis ST	A1T, A1L, A1V, A2T, A2L, A2V, HCT, HC PD, DAR-2, IR, ARTh, SP-A1
Wu De et al., 2020 [23]	100	6	4.91 ± 0.96 110 cap −4.88 ± 0.97 140 cap	SMILE 110 μm cap thickness SMILE 140 μm cap thickness	Corvis ST	Km-ant at 2-mm zone (D) Km-ant at 4-mm zone (D) Km-ant at 6-mm zone (D)
Wu Z et al., 2017 [24]	60 MILE, 64 SMILE	6	−5.54 ± 1.11 MILE −5.77 ± 1.55 SMILE	MILE 2 mm side cut SMILE 5 mm side cut	ORA	CH, CRF
Jun et al., 2021 [25]	91 120-μm cap group 59 140-μm cap group	6	−3.65 ± 1.42 120 μm cap −3.74 ± 1.58 140 μm cap	SMILE 120 μm cap thickness SMILE 140 μm cap thickness	Corvis ST	DA ratio, SP-A1 IIR, ARTh, SSI, bIOP
Vestergaard et al., 2014 [26]	70	6	−7.59 ± 0.97 FLEX, −7.56 ± 1.11 SMILE	FLEX, SMILE	ORA	CH, CRF

PRK, photorefractive keratectomy; t-PRK, transepithelial photorefractive keratectomy; PRK-MMC, photorefractive keratectomy with mitomycin C; LASIK, laser in situ keratomileusis; FS-LASIK, femtosecond LASIK; SMILE, small incision lenticule extraction; MILE, microincision lenticule extraction; LASEK, laser-assisted sub-epithelial keratectomy; PTA, percentage of tissue altered; ORA, Ocular Response Analyzer; CH, corneal hysteresis; CRF, corneal resistance factor; A1T, first applanation time; A1L, first applanation length; A1V, first applanation velocity; A2T, second applanation time; A2L, second applanation length; A2V, second applanation velocity, HCT, highest concavity time; HCPD, highest concavity peak distance; DA, deformation amplitude; IR, maximum inverse radius; IIR, integrated inverse radius; DAR-1, DA ratio of central and peripheral deflection is measured in a distance of 1 mm; DAR-2, DA ratio of central and peripheral deflection is measured in a distance of 2 mm; HC, highest concavity point; SPA1, stiffness parameter at the 1st applanation; AL, Applanation lengths; CVel, corneal velocities; ARTh, Ambrosio Rational Thickness horizontal; Pachy,¬ Pachyslope together with Corneal pachymetry; DCR, Dynamic Corneal Response; CBI, Corvis Biomechanical Index; TBI, tomographic and biomechanical index; IOP, intraocular pressure; bIOP, the biomechanical corrected intraocular pressure; DIC, digital image correlation;, HFU, high-frequency ultrasound imaging; dArcL, delta arc length; DLL, defection length; DLAr, defection area; Y, years; N/S, not specified.

**Table 2:** Characteristics of corneal biomechanical parameters assessed by ORA.

First Author	Refractive Procedure	Number of Eyes	Follow-Up Points/Study Groups	Preoperative CH (mmHg) Mean ± SD	Postoperative CH (mmHg) Mean ± SD	Difference in CH (mmHg)	Preoperative CRF (mmHg) Mean ± SD	Postoperative CRF (mmHg) Mean ± SD	Difference in CRF (mmHg)
Ryan et al., 2011 [10]	EPI-LASIK	51	6 M 12 M	10.22 ± 1.65	8.63 ± 1.31 8.53 ± 1.49	1.56 ± 0.86 1.62 ± 0.88	10.01 ± 1.80	7.77 ± 1.50 7.80 ± 1.66	2.18 ± 0.91 2.08 ± 1.03
Hwang et al., 2017 [12]	PRK, PRK-MMC, LASIK	194	12 M PRK PRK-MMC LASIK	N/S	9.7 ± 1.7 9.1 ± 1.7 10.1 ± 1.6	N/S N/S N/S	N/S	9.4 ± 1.9 9.2 ± 1.8 10.0 ± 1.8	N/S N/S N/S
Qazi et al., 2009 [13]	LASIK, LASEK	29	LASIK 6 M LASEK 6 M	10.00 ± 1.77 9.06 ± 1.56	8.57 ± 2.25 7.16 ± 1.99	1.71 ± 1.47 1.54 ± 1.38	9.87 ± 1.97 8.61 ± 1.76	7.35 ± 2.49 5.95 ± 2.41	3.05 ± 1.70 2.32 ± 1.93
Yu et al., 2019 [14]	SMILE LASEK	32	SMILE 3 Y LASEK 3 Y	10.5 ± 2.1 10.1 ± 1.3	8.7 ± 1.4 8.8 ± 1.5	N/S N/S N/S	11.1 ± 1.7 10.2 ± 1.6	7.4 ± 1.1 7.2 ± 1.7	N/S N/S N/S
Wu Di et al., 2014 [17]	SMILE, FS-LASIK	40	SMILE 6 M FS-LASIK 6 M	N/S	8.59 ± 1.00 8.11 ± 0.66	1.94± 0.82 2.34 ± 1.08	N/S	7.78 ± 1.03 6.94 ± 0.66	3.59 ± 0.91 4.29 ± 1.60
Elmohamady et al., 2018 [18]	LASIK, FS-LASIK, SMILE	103	LASIK 6 M 12 M 3Y FS-LASIK 6 M 12 M 3Y SMILE 6 M 12 M 3Y	10.82 ± 0.53 10.71 ± 0.47 10.58 ± 0.39	7.47 ± 0.54 7.45 ± 0.65 7.58 ± 0.71 7.58 ± 0.60 7.56 ± 0.44 7.60 ± 0.61 8.40 ± 0.37 8.37 ± 0.40 8.51 ± 0.51	N/S N/S N/S N/S N/S N/S N/S N/S N/S	10.19 ± 0.22 10.22 ± 0.20 10.21 ± 0.19	7.12 ± 0.76 7.11 ± 0.57 7.17 ± 0.68 7.21 ± 0.65 7.18 ± 0.59 7.25 ± 0.69 8.30 ± 0.48 8.29 ± 0.32 8.38 ± 0.59	N/S N/S N/S N/S N/S N/S N/S N/S N/S
Vanathi et al., 2020 [19]	FS-LASIK	80 group 1: PTA 23 to <27%; group 2: 27 to <33%; group 3: 33 to <40%	group 1 6 M group 2 6 M group 3 6 M	11.06 ± 1.36 10.54 ± 2.33 10.15 ± 1.47	9.08 ± 0.91 8.39 ± 1.11 7.42 ± 0.86	18% 20% 26%	11.85 ± 1.99 10.23 ± 1.42 10.23 ± 1.35	8.63 ± 0.99 7.83 ± 1.14 6.81 ± 1.33	27% 23% 33%
Agca et al., 2014 [20]	SMILE, FS-LASIK	60	SMILE 6 M FS-LASIK 6 M	10.89 ± 1.79 11.00 ± 1.53	8.95 ± 1.47 9.02 ± 1.27	1.94 ± 1.52 1.98 ± 1.50	10.73 ± 1.71 10.76 ± 1.45	7.77 ± 1.37 8.07 ± 1.26	2.96 ± 1.69 2.69 ± 1.44
Kamiya et al., 2009 [21]	LASIK	36	LASIK 6 M	10.68 ± 1.7	8.9 ± 1.5	N/S	10.0 ± 1.7	7.7 ± 1.6	N/S
Wu Z et al., 2017 [24]	MILE (2 mm side cut), SMILE (5 mm side cut)	124	MILE 6 M SMILE 6 M	10.03 ± 1.31 10.11 ± 0.96	8.30 ± 0.77 8.15 ± 0.77	N/S N/S	10.13 ± 1.38 10.50 ± 0.97	6.93 ± 1.05 6.95 ± 0.96	N/S N/S
Vestergaard et al., 2014 [26]	FLEX, SMILE	70	6 M FLEX SMILE	10.8 ± 1.7 11.0 ± 1.7	8.0 ± 1.1 7.8 ± 1.3	2.7 ± 1.3 3.3 ± 1.2	10.9 ± 1.8 10.9 ± 1.9	6.4 ± 1.4 6.4 ± 1.4	4.5 ± 1.2 4.6 ± 1.2

EPI-LASIK, epithelial laser in-situ keratomileusis; PRK, photorefractive keratectomy; PRK-MMC, photorefractive keratectomy with mitomycin C; LASIK, laser in situ keratomileusis; LASEK, laser-assisted sub-epithelial keratectomy; SMILE, small incision lenticule extraction; FS-LASIK, femtosecond LASIK; MILE, microincision lenticule extraction; FLEX, femtosecond lenticule extraction; ORA, Ocular Response Analyzer; CH, corneal hysteresis; CRF, corneal resistance factor; W, weeks; M, months; Y, years; N/S, not specified.

**Table 3:** Characteristics of corneal biomechanics assessed by Corvis- ST.

First Author	Refractive Procedure	Number of Eyes	Follow-Up Points (Months)	Parameter	Preoperative	Postoperative	Difference
Xin et al., 2022 [11]	t-PRK, FS-LASIK, SMILE	227	6 t-PRK, LM group/ HM group				Pre vs post
				SP-A1	96.8 ± 16.6/99.2 ± 17.0	74.5 ± 19.4/68.0 ± 17.1	−27.40 ± 16.91/N/S
				IIR (mm^−1^)	8.69 ± 1.04/8.49 ± 0.78	10.64 ± 0.95/11.29 ± 0.94	2.40 ± 0.94/N/S
				DA (mm)	1.06 ± 0.09/1.06 ± 0.07	1.15 ± 0.11/1.17 ± 0.10	0.101 ± 0.086/N/S
				DAR-2 (mm)	4.85 ± 0.54/4.75 ± 0.41	5.44 ± 0.58/5.72 ± 0.46	0.79 ±0.55/N/S
			FS-LASIK LM group/ HM group	SP-A1	96.3 ± 12.6/103.4 ± 16.4	68.3 ± 13.5/64.8 ± 14.8	−34.15 ± 13.17/N/S
				IIR (mm^−1^)	8.68 ± 1.08/8.16 ± 1.07	11.03 ± 1.00/11.38 ± 0.97	2.85 ± 0.96/N/S
				DA (mm)	1.04 ± 0.08/1.04 ± 0.09	1.16 ± 0.08/1.19 ± 0.07	0.134 ± 0.057/N/S
				DAR-2 (mm)	4.79 ± 0.45/4.48 ± 0.47	5.92 ± 0.64/5.86 ± 0.51	1.28 ± 0.53/N/S
			SMILE LM group/ HM group	SP-A1	99.1 ± 13.9/99.1 ± 13.9	70.0 ± 12.4/70.0 ± 12.4	−32.40 ± 10.42/N/S
				IIR (mm^−1^)	8.72 ± 0.90/8.52 ± 0.85	11.16 ± 1.01/11.72 ± 0.83	2.84 ± 1.03/N/S
				DA (mm)	1.06 ± 0.09/1.08 ± 0.08	1.18 ± 0.07/1.19 ± 0.06	0.118 ± 0.063/N/S
				DAR-2 (mm)	4.83 ± 1.03/4.63 ± 0.37	5.77 ± 0.49/5.97 ± 0.46	1.15 ± 0.83/N/S
Hashemi et al., 2017 [15]	FS-LASIK PRK + MMC	60	6 FS-LASIK/ PRK-MMC	IOP (mm Hg)	N/S	13.42 ± 1.39/11.41 ± 1.25	N/S
				bIOP (mm Hg)	N/S	18.65 ± 1.77/17.97 ± 1.46	N/S
				A1T (ms)	N/S	6.86 ± 0.22/6.55 ± 0.21	N/S
				A2T (ms)	N/S	21.13 ± 0.24/21.38 ± 0.19	N/S
				A1L (mm)	N/S	1.76 ± 0.41/1.75 ± 0.33	N/S
				A2L (mm)	N/S	1.59 ± 0.51/1.42 ± 0.50	N/S
				A1V (m/s)	N/S	0.11 ± 0.04/0.12 ± 0.04	N/S
				A2V (m/s)	N/S	−0.49 ± 0.10/−0.41 ± 0.16	N/S
				HCT (ms)	N/S	16.31 ± 0.13/16.36 ± 0.29	N/S
				DA	N/S	0.95 ± 0.34/1.07 ± 0.10	N/S
				PD	N/S	4.33 ± 1.30/4.59 ± 1.20	N/S
				HCR (mm)	N/S	5.71 ± 0.25/5.47 ± 0.46	N/S
Yang et al., 2020 [16]	LASIK	23	12	IR (mm^−1^)	N/S	0.036	N/S
				DAR-2 (mm)	N/S	1.123	N/S
				Pachy Slope	N/S	72.917	N/S
				DAR-1 (mm)	N/S	0.110	N/S
				ARTh	N/S	−382.214	N/S
				IR (mm^−1^)	N/S	2.212	N/S
				SP-A1	N/S	−27.459	N/S
				CBI	N/S	0.948	N/S
He et al., 2022 [22]	SMILE, FS-LASIK	50	6 SMILE/FS-LASIK	A1T (ms)	7.65 ± 0.36/7.63 ± 0.36	7.09 ± 0.19/7.12 ± 0.20	−0.03 (−0.08, 0.02)
				A1L (mm)	2.14 ± 0.34/2.14 ± 0.36	1.94 ± 0.19/1.90 ± 0.21	0.03 (−0.05, 0.11)
				A1V (m/s)	0.15 ± 0.02/0.15 ± 0.02	0.15 ± 0.01/0.16 ± 0.02	−0.005(−0.009, −0.001)
				A2T (ms)	22.23 ± 0.35/22.30 ± 0.38	22.82 ± 0.32/22.82 ± 0.35	−0.003 (−0.09, 0.09)
				A2L (mm)	2.09 ± 0.50/2.03 ± 0.40	1.35 ± 0.36/1.40 ± 0.37	−0.05 (−0.20, 0.11)
				A2V (m/s)	−0.26 ± 0.04/−0.26 ± 0.03	−0.28 ± 0.02/−0.28 ± 0.03	−0.004 (−0.013, 0.004)
				HCT (ms)	17.36 ± 0.48/17.43 ± 0.48	17.38 ± 0.43/17.38 ± 0.96	−0.005 (−0.29, 0.28)
				HCR	7.59 ± 0.92/7.71 ± 1.12	6.13 ± 0.38/6.05 ± 0.39	0.08 (−0.02, 0.18)
				HCPD	5.05 ± 0.30/5.09 ± 0.30	5.46 ± 0.16/5.43 ± 0.21	0.03 (−0.01, 0.08)
				DAR	4.19 ± 0.41/4.29 ± 0.54	5.57 ± 0.82/5.49 ± 0.48	0.93 (−2.96, 1.11)
				IR (mm^−1^)	7.89 ± 1.05/7.87 ± 1.02	11.14 ± 0.65/11.28 ± 0.86	0.14 (−0.12, 0.30)
				ARTh	538.49 ± 94.68/525.63 ± 99.28	128.95 ± 13.38/118.18 ± 14.94	9.78 (6.27, 13.29)
				SP-A1	117.67 ± 22.31/114.62 ± 17.16	81.27 ± 15.56/76.64 ± 16.48	4.62 (1.76, 7.49)
				CBI	0.10 ± 0.19/0.11 ± 0.19	0.03 ± 0.12/0.05 ± 0.18	−0.02 (−0.08, 0.04)
				SSI	0.89 ± 0.11/0.89 ± 0.13	0.83 ± 0.09/0.90 ± 0.28	−0.08 (−0.16, −0.008)
Wu De et al., 2020 [23]	SMILE 110-μm cap SMILE 140-μm cap	100	6 110-μm cap/140-μm cap	A1L (mm)	N/S	N/S	0.347 ± 0.462/0.224 ± 0.39
				A1V (m/s)	N/S	N/S	−0.01/−0.01
				A1T (ms)	N/S	N/S	0.415/0.53
				A2L (mm)	N/S	N/S	0.357 ± 0.403/0.35 ± 0.492
				A2V (m/s)	N/S	N/S	0.010/0.005
				A2T (ms)	N/S	N/S	−0.283 ± 0.339/−0.411 ± 0.402
				DPD (mm)	N/S	N/S	−0.297 ± 0.208/−0.323 ± 0.248
				HCR (mm)	N/S	N/S	0.885/1.080
				DA (mm)	N/S	N/S	−0.078 ± 0.072/−0.104 ± 0.084
				bIOP (mm Hg)	N/S	N/S	1.426 ± 1.519/1.912 ± 1.558
				IR (mm^−1^)	N/S	N/S	−2.220 ± 0.71/−2.754 ± 0.728
				SP-A1	N/S	N/S	28.188 ± 12.012/29.836 ± 10.959
				DAR	N/S	N/S	−1.12 ± 0.36/−1.24 ± 0.389
Jun et al., 2021 [25]	SMILE 120-μm cap SMILE 140-μm cap	150	6 120-μm cap/140-μm cap	DAR	4.30 ± 0.37/4.24 ± 0.33	4.8 ± 0.51/5.57 ± 0.45	1.18 ± 0.40/1.33 ± 0.36
				SP-A1	115.17 ± 10.95/117.99 ± 11.14	93.02 ± 10.74/93.67 ± 10.87	−22.35 ± 9.60/−24.32 ± 11.08
				IIR (mm^−1^)	8.17 ± 0.79/8.11 ± 0.90	10.53 ± 1.02/10.81 ± 1.18	2.35 ± 0.75/2.71 ± 0.93
				ARTh	462.00 ± 93.82/487.12 ± 107.85	209.98 ± 54.87/208.16 ± 64.01	−252.32 ± 86.54/−278.97 ± 92.77
				SSI	1.02 ± 0.13/1.03 ± 0.15	0.88 ± 0.14/0.86 ± 0.13	−0.14 ± 0.15/−0.17 ± 0.10
				bIOP (mm Hg)	15.89 ± 1.19/16.25 ± 1.33	15.77 ± 1.17/16.06 ± 1.20	−0.13 ± 0.85/−0.19 ± 0.75

t-PRK, transepithelial photorefractive keratectomy; FS-LASIK, femtosecond LASIK; SMILE, small incision lenticule extraction; PRK-MMC, photorefractive keratectomy with mitomycin C; LASIK, laser in situ keratomileusis; LM, low myopia; HM, high myopia; A1T, first applanation time; A1L, first applanation length; A1V, first applanation velocity; A2T, second applanation time; A2L, second applanation length; A2V, second applanation velocity, HCT, highest concavity time; HCPD, highest concavity peak distance; DA, deformation amplitude; IR, maximum inverse radius; IIR, integrated inverse radius; DAR-1, DA ratio of central and peripheral deflection is measured in a distance of 1 mm; DAR-2, DA ratio of central and peripheral deflection is measured in a distance of 2 mm; HC, highest concavity point; SPA1, stiffness parameter at the 1st applanation; AL, Applanation lengths; CVel, corneal velocities; ARTh, Ambrosio Rational Thickness horizontal; Pachy, Pachyslope together with Corneal pachymetry; DCR, Dynamic Corneal Response; CBI, Corvis Biomechanical Index; TBI, tomographic and biomechanical index; IOP, intraocular pressure; bIOP, the biomechanical corrected intraocular pressure; DIC, digital image correlation; HFU, high frequency ultrasound imaging; dArcL, delta arc length; DLL, defection length; DLAr, defection area; LM, low myopia; HM, high myopia; N/S, not specified, pre, preoperative; post, postoperative.

## Data Availability

All the materials and information will be available upon request by e-mail to the corresponding author.
